# The Roles of Imprinted *SLC22A18* and *SLC22A18AS* Gene Overexpression Caused by Promoter CpG Island Hypomethylation as Diagnostic and Prognostic Biomarkers for Non-Small Cell Lung Cancer Patients

**DOI:** 10.3390/cancers12082075

**Published:** 2020-07-27

**Authors:** José Francisco Noguera-Uclés, Laura Boyero, Ana Salinas, Juan Antonio Cordero Varela, Johana Cristina Benedetti, Reyes Bernabé-Caro, Amparo Sánchez-Gastaldo, Miriam Alonso, Luis Paz-Ares, Sonia Molina-Pinelo

**Affiliations:** 1 Institute of Biomedicine of Seville (IBiS) (HUVR, CSIC, Universidad de Sevilla), 41013 Seville, Spain; josnogucl@alum.us.es (J.F.N.-U.); lboyero-ibis@us.es (L.B.); asalinas-ibis@us.es (A.S.); jacordero-ibis@us.es (J.A.C.V.); johanac.benedetti.sspa@juntadeandalucia.es (J.C.B.); reyesbernab@yahoo.es (R.B.-C.); asanchezgastaldo@gmail.com (A.S.-G.); miriamag3@hotmail.com (M.A.); 2 Medical Oncology Department, Hospital Universitario Virgen del Rocío, 41013 Seville, Spain; 3 Centro de Investigación Biomédica en Red de Cáncer (CIBERONC), 28029 Madrid, Spain; lpazaresr@seom.org; 4 H12O-CNIO Lung Cancer Clinical Research Unit, Instituto de Investigación Hospital 12 de Octubre & Centro Nacional de Investigaciones Oncológicas (CNIO), 28029 Madrid, Spain; 5 Medical Oncology Department, Hospital Universitario 12 de Octubre, 28041 Madrid, Spain

**Keywords:** NSCLC, *SLC22A18*, *IMPT1*, *TSSC5*, *SLC22A18AS*, genomic imprinting, prognosis, diagnostic, biomarkers

## Abstract

Genomic imprinting is a process that involves one gene copy turned-off in a parent-of-origin-dependent manner. The regulation of imprinted genes is broadly dependent on promoter methylation marks, which are frequently associated with both oncogenes and tumor suppressors. The purpose of this study was to assess the DNA methylation patterns of the imprinted solute-carrier family 22 member 18 (*SLC22A18)* and *SLC22A18* antisense *(SLC22A18AS)* genes in non-small cell lung cancer (NSCLC) patients to study their relevance to the disease. We found that both genes were hypomethylated in adenocarcinoma and squamous cell carcinoma patients. Due to this imprinting loss, *SLC22A18* and *SLC22A18AS* were found to be overexpressed in NSCLC tissues, which is significantly more evident in lung adenocarcinoma patients. These results were validated through analyses of public databases of NSCLC patients. The reversed gene profile of both genes was achieved in vitro by treatment with ademetionine. We then showed that high *SLC22A18* and *SLC22A18AS* expression levels were significantly associated with worsening disease progression. In addition, low levels of *SLC22A18AS* were also correlated with better overall survival for lung adenocarcinoma patients. We found that SLC22A18 and SLC22A18AS knockdown inhibits cell proliferation in vitro. All these results suggest that both genes may be useful as diagnostic and prognostic biomarkers in NSCLC, revealing novel therapeutic opportunities.

## 1. Introduction

Epigenetic studies have revealed inheritable and reversible changes in the genome, which, without modifying nucleic acid sequences, alter all key DNA-dependent processes, such as replication, repair, recombination and transcription [1]. Epigenetic mechanisms include DNA methylation, histone modifications, nucleosome remodeling, and alterations in microRNA expression [2]. Currently, one of the most widely studied epigenetic processes is DNA methylation, in which a methyl group is added at the fifth carbon position of cytosine residues in the context of a CpG dinucleotide [3,4]. The CpG dinucleotides are not distributed uniformly across the human genome; they tend to cluster into small dense sequences known as CpG islands, which make up 1% of the full genome sequence [2]. CpG islands are enriched in gene promoter sequences, playing key roles in the regulation of gene expression under physiological conditions. However, aberrations to DNA methylation contribute to the development and progression of diseases such as cancer [5]. For instance, hypermethylation in the promoter region of tumor suppressor genes reduces their expression, and in contrast, cancer-linked DNA hypomethylation is associated with the overexpression of oncogenes, whereas methylation in a transcribed region has various effects on gene expression in tumor cells [4].

Over the past decade, there has been increasing evidence showing the relevant role of epigenetic markers in non-small cell lung cancer (NSCLC); these tumors are the deadliest tumors worldwide and represent 80% of all types of lung cancer, with the other 20% being small cell lung cancer (SCLC). NSCLC can be histologically subclassified into squamous cell carcinoma (SCC) (40%), adenocarcinoma (30%) and large cell carcinoma (10%). The detection of lung cancer at an early stage is correlated with a better prognosis; however, unfortunately, this occurs only in 15–20% of diagnosed patients [6]. The relationship between tobacco and lung cancer is well known, with approximately 80% of cases directly related to smoking in Western countries [7]. Smoking produces chronic inflammation and an increase in reactive oxygen species, leading to alterations in DNA methylation in defined nuclear positions and chromosome domains [8]. It is notable that, although most lung cancer patients are smokers, only a minority of lifetime smokers develop the disease. This finding suggests that lung carcinogenesis is strongly related to genetic and/or epigenetic susceptibility [4].

In general, NSCLC shows global hypomethylation [9,10], which is associated with genomic instability. This global demethylation is generally an early event, occurring during tumor initiation and progression, and its significance depends the part of the genome and the genes affected [5,11]. In addition, hypermethylation also occurs, but specifically on tumor suppressor genes, such as *P16*. In lung cancer, hypermethylated genes are involved in functions such as DNA repair, apoptosis, cell cycle, cell adhesion and invasion, and transcription regulation [2]. In summary, gene methylation status has shown potential diagnostic, prognostic and treatment-response predictive value, which makes it a promising epigenetic tumor biomarker. The majority of the genes deregulated due to DNA methylation are inherited from two functionally equivalent parental copies. However, there is a small subset in which one allele is turned off through an epigenetic mechanism in a parent-of-origin-dependent manner, known as genomic imprinting [12]. Loss of genomic imprinting is often associated with oncogenes or tumor suppressor genes. For example, the imprinted *P53* and *IGF2* genes have been reported to be tumor suppressor genes and oncogenes, respectively [12,13]. Alternatively, overexpression of some imprinted miRNAs has been proposed as a regulator of tumor suppressor genes [14]. Thus, imprinted messenger RNA aberrations seem to play a key role in cancer. However, there are still many imprinted genes whose underlying role in NSCLC remains unknown. Therefore, we analyzed whether the methylation status of imprinted solute carrier (SLC) transporters, namely, *SLC22A18* and its antisense gene (*SLC22A18AS*), have clinical significance in patients with NSCLC.

The *SLC22A18* and *SLC22A18AS* genes are a sense-antisense pair located on chromosomal segment 11p15.5, an imprinted region that is 1.25 Mb long with a total of 21 genes [15]. These genes partially overlap in divergent orientations such that the first exon of *SLC22A18AS* shares 31 bp with the second exon of *SLC22A18* [16]. While the sense gene (*SLC22A18*) is a 10 transmembrane domain member of a family of polyspecific transporters and multidrug resistance genes and is expressed in the liver and kidney, little is known about its antisense partner (*SLC22A18AS*). There is still no consensus among the scientific community on the role of *SLC22A18* in the tumor process, since, depending on the type of cancer, it will have either a tumor suppressor function or be a promoter of tumorigenesis [17]. Due to the importance of genomic imprinting in correct gene expression and the role that epigenetic imbalances play in tumorigenesis and its progression, the *SLC22A18* and *SLC22A18AS* genes may be potential biomarkers of NSCLC. However, little is known about the function(s) of these genes in the development and prognosis of this disease. Therefore, the purpose of this study was to elucidate the roles of the *SLC22A18* and *SLC22A18AS* genes in NSCLC. To this end, we analyzed the transcriptional regulation mechanisms of both imprinted genes and their potential uses as prognostic markers in patients with NSCLC.

## 2. Results

### 2.1. CpG Promoter Methylation Status of the Imprinted SLC22A18 and SLC22A18AS Genes in NSCLC Patients

To evaluate the potential role of the *SLC22A18* and *SLC22A18AS* genes in lung cancer, we analyzed the DNA methylation pattern of its promoter in human lung tissues (Figure S1). The DNA methylation profile of *SLC22A18* and *SLC22A18AS* was studied in tumor samples and compared to nontumor tissue in the first patient cohort using an Illumina Infinium Human Methylation 450 BeadChip. Significant differences were identified for the *SLC22A18* (*p* < 0.0001) and *SLC22A18AS* (*p* < 0.0001) genes in tumors in relation to the nontumor tissue. Both genes were hypomethylated in NSCLC patients. The hypomethylation status was consistently observed for patients subclassified into the two main histological subtypes of NSCLC. We found that adenocarcinoma patients showed significantly lower methylation levels of both the *SLC22A18* and *SLC22A18AS* genes than the SCC patients. In addition, methylation levels for both genes were significantly lower in adenocarcinoma with respect to SCC subtype (*p* < 0.01). To validate these findings, we analyzed the differential methylation levels of both genes from an independent cohort of NSCLC patients identified in The Cancer Genome Atlas (TCGA) database. Significant differences in the methylation levels between tumor and nontumor tissues were observed for both genes (*p* < 0.001). Then, we analyzed the methylation levels according to the disease stage, comparing late stage (III–IV) and early stage (I–II) samples. However, we did not find significant differences in methylation levels in tissues of different tumor stages. 

**Figure 1:**
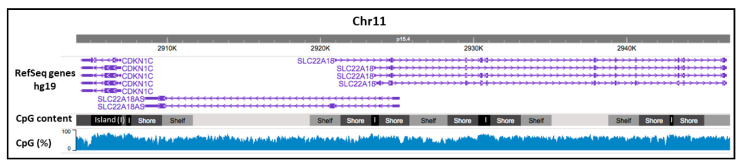
CpG density in the *SLC22A18* and *SLC22A18AS* genes. This image illustrates the CpG content level and CpG percentage in both genes. Purple arrows represent the exact position of both genes on Chr.11p15.5. CpG-rich regions (CpG islands, “I”) are highlighted in black; regions up to 2 kb from the CpG island (shores) are marked in dark gray; regions from 2 to 4 kb from the CpG island (shelves) are highlighted in intermediate gray; and the rest of the gene is colored in light gray. The blue histogram represents the CpG percentage in this chromosomal region.

To explore the CpG density in the 11p15.5 chromosomic region, which carries the imprinted *SLC22A18* and *SLC22A18AS* genes, we generated an epigenome map using the WashU Epigenome Browser (Figure 1). A high CpG rate was observed throughout the genomic sequence of both genes (>50%). We found five CpG islands in the chromosome region, one of which was located in the promoter region of the *SLC22A18* gene and its antisense gene. CpG shores and CpG shelves flanked all the CpG islands, whose CpG density was progressively lower away from the CpG islands.

### 2.2. Expression Levels of the Imprinted SLC22A18 and SLC22A18AS Genes in NSCLC Tissue

**Figure 2:**
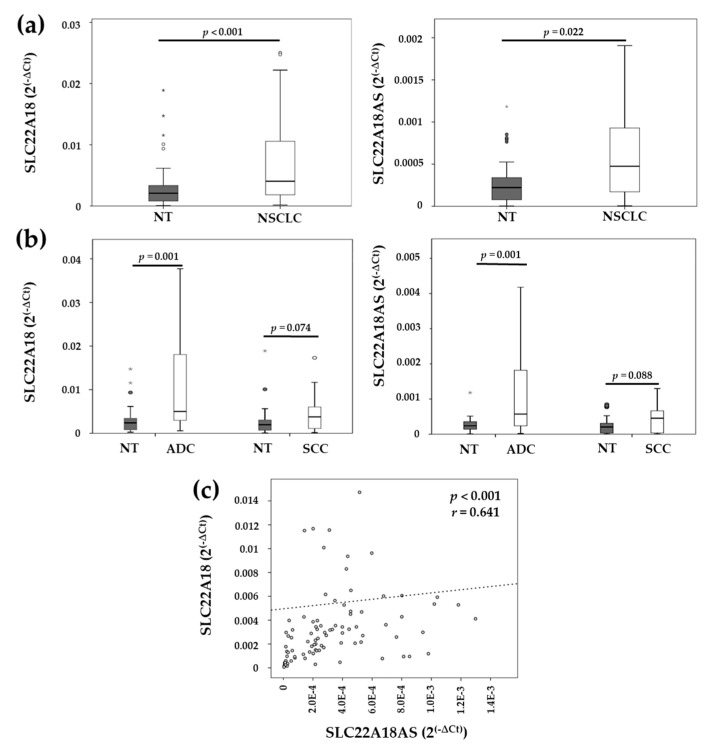
Expression levels of the *SLC22A18* and *SLC22A18AS* genes in the tumor and matched nontumoral samples from patients with lung cancer. (**a**) Comparison of the *SLC22A18* and *SLC22A18AS* gene expression levels in the NSCLC tissue. (**b**) Comparison of the expression levels of both genes according to histological subtypes. NSCLC: non-small cell lung cancer; ADC: adenocarcinoma; SCC: squamous cell carcinoma; NT: nontumor. Boxes show the interquartile range (IQR) and median (thick line); whiskers indicate the range. Outliers > 1.5 × IQR but < 3 × IQR from the nearest edge of the box are represented by open circles and those > 3 × IQR from the nearest edge of the box are represented by * symbols. (**c**) Spearman’s correlation of the *SLC22A18* and *SLC22A18AS* expression levels.

To study the effect of *SLC22A18* and *SLC22A18AS* methylation status on transcriptional regulation, we analyzed the expression levels of both genes by qPCR in the second cohort of NSCLC patients. The results confirm an inverse association between the methylation status and the expression levels of both genes, which were significantly increased for *SLC22A18* (*p* < 0.001) and *SLC22A18AS* (*p* = 0.022) in the NSCLC tissue compared to the matched nontumor lung tissue (Figure 2a). Next, we analyzed their expression levels according to histological subtypes. We found that *SLC22A18* and *SLC22A18AS* were significantly overexpressed in adenocarcinoma (*p* = 0.001) relative to the nontumor lung tissue. On the other hand, no significant differences were found for these genes in the SCC tissue compared with the expression in the nontumor tissue; however, an upward trend was observed (*p* = 0.074 and *p* = 0.088, respectively) (Figure 2b). We also compared the expression levels of the *SLC22A18* and *SLC22A18AS* genes in tissues of the main histological subtypes of NSCLC. Both genes showed higher expression levels in the lung adenocarcinoma samples than they did in the SCC samples, but these differences did not reach a level of significance. 

Owing to *SLC22A18* and *SLC22A18AS* are in the same cluster, we analyzed whether their expression was correlated in the NSCLC patient samples. We found a significant positive correlation between the *SLC22A18* and *SLC22A18AS* genes (*r* = 0.641; *p* < 0.001) (Figure 2c).

### 2.3. Validation of the Expression Profiles of the SLC22A18 and SLC22A18AS Genes in the NSCLC Samples in Public Databases

**Figure 3:**
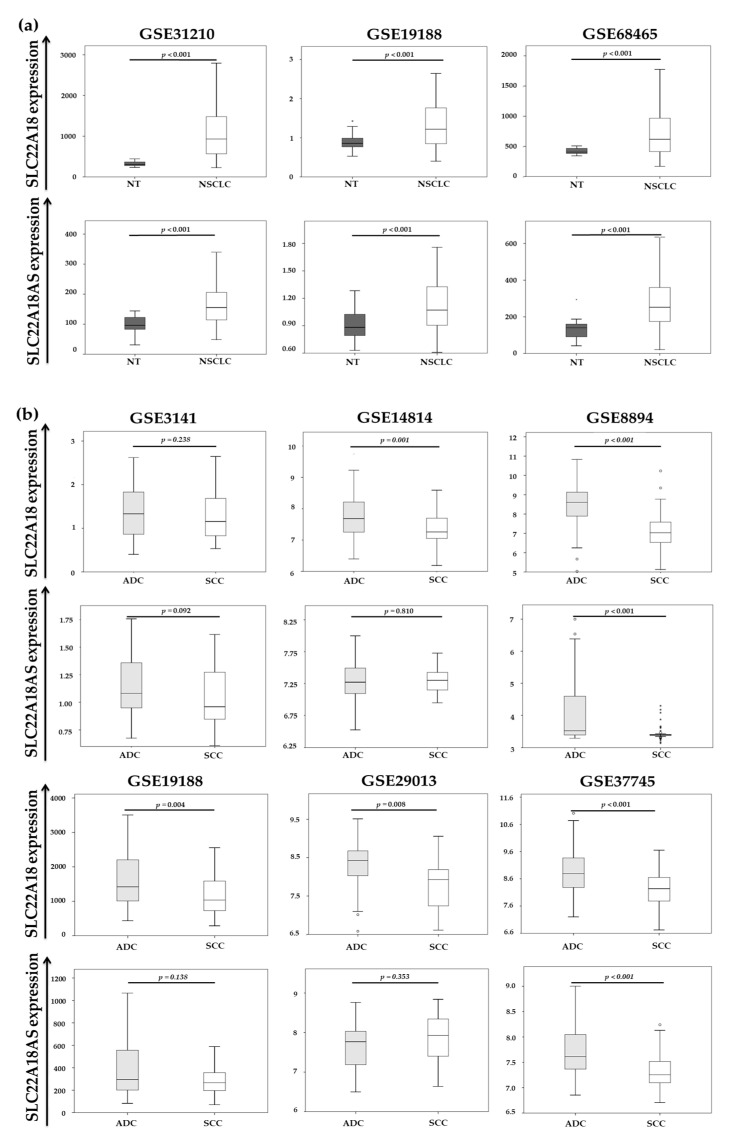
Analysis of the *SLC22A18* and *SLC22A18AS* expression levels from NCBI Gene Expression Omnibus (GEO) datasets. (**a**) Differential expression of the *SLC22A18* and *SLC22A18AS* genes in the NSCLC tissue and nontumor tissue. (**b**) Comparison of *SLC22A18* and *SLC22A18AS* expression in the lung adenocarcinoma and SCC tissue. NSCLC: non-small cell lung cancer; ADC: adenocarcinoma; SCC: squamous cell carcinoma; and NT: nontumor. Boxes show the interquartile range (IQR) and median (thick line); whiskers indicate the range. Outliers > 1.5 × IQR but < 3 × IQR from the nearest edge of the box are represented by open circles and those > 3 × IQR from the nearest edge of the box are represented by * symbols.

We expanded the expression analysis to eight different lung cancer datasets (GSE3141, GSE8894, GSE14814, GSE19188, GSE29013, GSE31210 GSE37745 and GSE68465) by obtaining the expression data for *SLC22A18* and *SLC22A18AS* (Figure 3 and Figure S2). In datasets where both nontumor and tumor lung tissue expression data were available (GSE31210, GSE19188 and GSE68465), *SLC22A18* and *SLC22A18AS* expression was analyzed, and overexpression of both genes was found in NSCLC tissues (*p* < 0.001) in all three of these datasets (Figure 3a). One of these datasets, GSE19188, also allowed us to compare the expression levels of *SLC22A18* and *SLC22A18AS* for each histological NSCLC subtype (lung adenocarcinoma and SCC) independently with respect to the nontumor tissues. The results are similar to those previously observed for the second patient cohort analyzed, showing *SLC22A18* and *SLC22A18AS* overexpression in lung adenocarcinoma (*p* < 0.001 for both genes) and SCC (*p* = 0.009 and *p* = 0.048, respectively) with respect to the nontumoral tissues.

We also compared the expression levels of both genes in the two main histological subtypes of NSCLC (adenocarcinoma and SCC). The analyses were performed in those databases that had expression data for both genes differentiated specifically by histological subtypes (GSE3141, GSE14814, GSE8894, GSE19188, GSE29013, and GSE37745). Most of the datasets showed significantly higher expression of *SLC22A18* and *SLC22A18AS* in the adenocarcinoma relative to the SCC subtype (Figure 3b).

**Figure 4:**
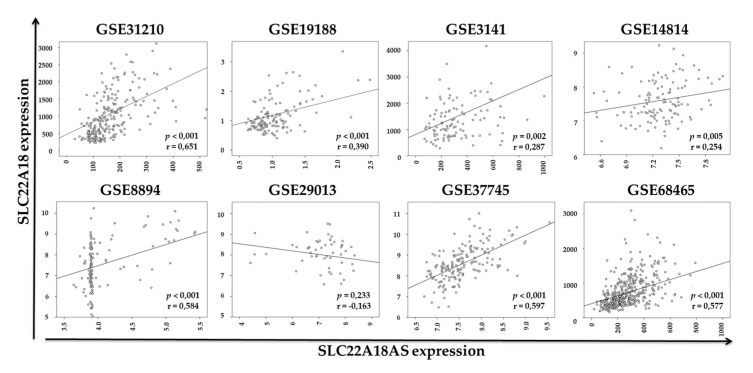
Spearman’s correlation analysis of *SLC22A18* and *SLC22A18AS* expression based on different datasets.

After confirming that NSCLC patients had aberrant expression of the *SLC22A18* and *SLC22A18AS* genes, as identified through public databases, we investigated whether there was a genetic correlation between both genes, as observed in the second patient cohort. The Spearman’s correlation analysis showed a significant positive association for all except one of the consulted datasets (Figure 4).

### 2.4. Rescue of DNA Methylation Status of the SLC22A18 and SLC22A18AS Genes In Vitro by Treatment with Ademetionine 

**Figure 5:**
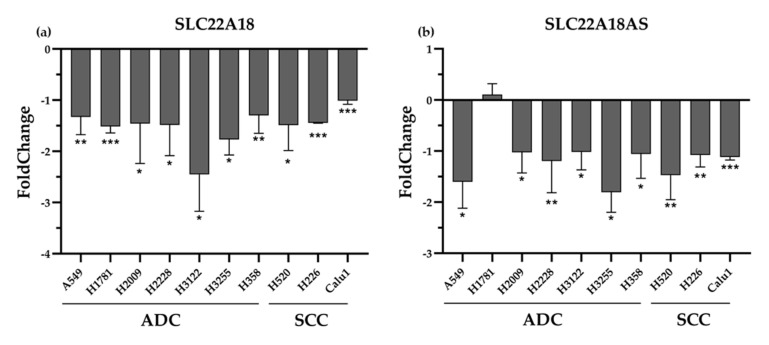
Effect of ademetionine supplementation on the expression of *SLC22A18* and *SLC22A18AS.* Real-time PCR (qPCR) was conducted to quantify *SLC22A18* (**a**) and *SLC22A18AS* (**b**) mRNA expression relative to Dimethyl Sulfoxide (DMSO)-treated controls at 24 h after ademetionine treatment (200 μM). ADC: adenocarcinoma; SCC: squamous cell carcinoma. *** *p*-value < 0.0001; ** *p*-value < 0.001; and * *p*-value < 0.05.

After observing, on the one hand, a DNA hypomethylation state of *SLC22A18* and *SLC22A18AS* and, on the other, an increase in the expression of both genes in NSCLC tissues, we hypothesized that it was due to a direct relationship. As a proof of concept, we examined whether ademetionine, a universal and ubiquitous methyl donor that increases DNA methylation, could decrease the expression of these genes in a panel of 10 NSCLC cell lines (seven adenocarcinoma and three SCC). After 24 h of treatment, *SLC22A18* and *SLC22A18AS* expression levels were significantly rescued for both histological subtypes (Figure 5). Thus, the methylation status influences in the expression of the imprinted *SLC22A18* and *SLC22A18AS* genes in NSCLC.

### 2.5. Prognostic Roles of the Imprinted SLC22A18 and SLC22A18AS Genes for NSCLC Patients

**Figure 6:**
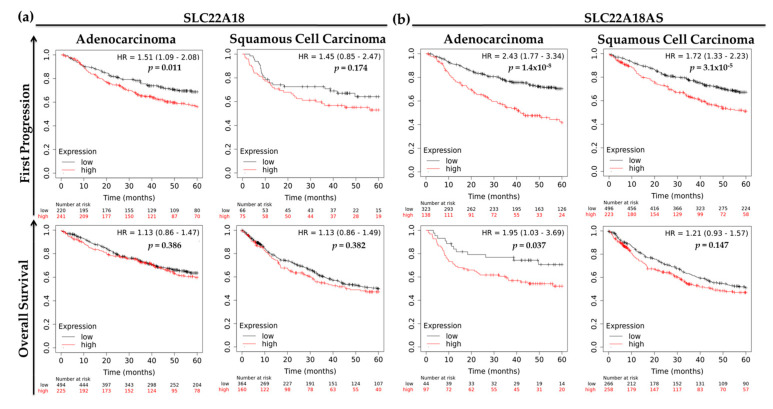
Five-year clinical outcomes for the two main histological subtypes of NSCLC from the Cancer Biomedical Informatics Grid (caBIG), GEO and The Cancer Genome Atlas (TCGA) repositories according to the expression levels of (**a**) SLC22A18 (probeset 204981_at) and (**b**) SLC22A18AS (probeset 206097_at). HR: hazard ratio.

To evaluate whether *SLC22A18* and *SLC22A18AS* expression levels were associated with clinical outcomes for patients with the main histological subtypes of NSCLC, we analyzed *SLC22A18* and *SLC22A18AS* expression levels by first progression of disease and overall survival data using the KM Plotter website [18]. For lung adenocarcinoma patients, we found that a higher expression of *SLC22A18* and *SLC22A18AS* was significantly associated with worsening disease progression (hazard ratio (HR) = 1.51, 95% confidence interval (CI) = 1.09–2.08, *p* = 0.011; and HR = 2.43, 95% CI = 1.77–3.34, *p* < 0.001, respectively). Then, low levels of *SLC22A18AS* were also associated with a better overall survival (HR = 1.95, 95% CI = 1.03–3.69, *p* = 0.037) (Figure 6a). However, we found nonsignificant differences for the *SLC22A18* expression levels with respect to overall survival in the patients with lung adenocarcinoma. In the case of SCC, the trend was similar, and lower expression of both genes trended with a better clinical outcome. Nevertheless, these differences were significant only for the time to first disease progression (HR = 1.72, 95% CI = 1.33–2.23, *p* < 0.001) (Figure 6b).

### 2.6. SLC22A18 and SLC22A18AS Knockdown Impairs Tumor Cell Proliferation

**Figure 7:**
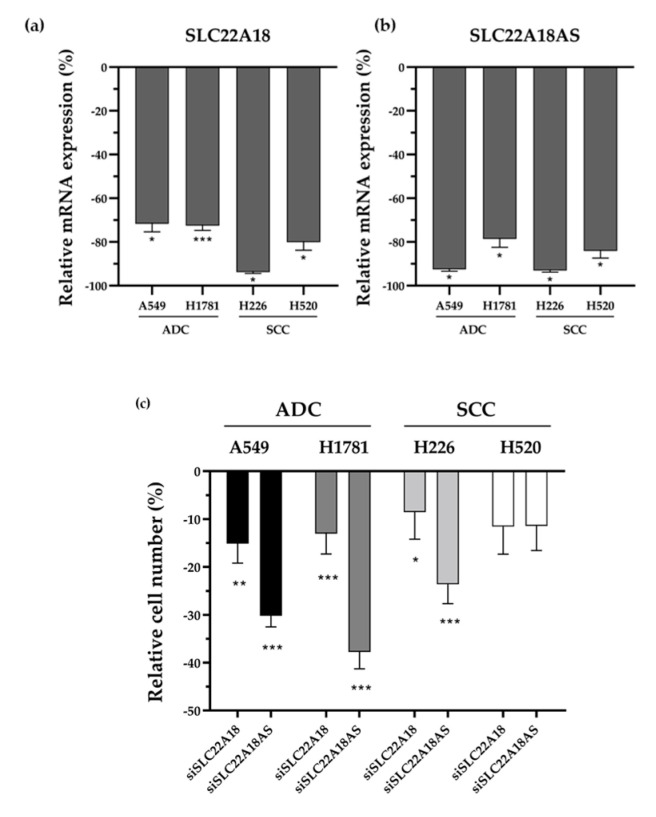
*SLC22A18* and *SLC22A18AS* knockdown inhibit cell proliferation. qPCR analysis of *SLC22A18* (**a**) and *SLC22A18AS* (**b**) mRNA expression at 72 h post-transfection of each siRNA relativized to negative control (**c**) Relative cell proliferation assay in *SLC22A18* and *SLC22A18AS* knockdown versus negative control after specific transfection. ADC: adenocarcinoma; SCC: squamous cell carcinoma. siSLC22A18: knockdown siRNA against SLC22A18; siSLC22A18AS: knockdown siRNA against SLC22A18AS. *** *p*-value < 0.0001; ** *p*-value < 0.001; and * *p*-value < 0.05.

The observed association between *SLC22A18* and *SLC22A18AS* gene overexpression and impaired patient survival made us hypothesize about whether there was a functional relationship between the regulation of these genes and tumorigenesis. For this, we tested the proliferative activity of *SLC22A18* and *SLC22A18AS* genes and their functional effect on lung cancer regulation by small interfering RNA (siRNA) knockdown of each gene in adenocarcinoma and SCC cell lines. Effective downregulation of each gene expression was confirmed by qPCR (Figure 7a,b). The silenced expression of both genes significantly impaired cell proliferation in almost all tested cell lines of both histological subtypes (Figure 7c). Only one squamous cell carcinoma cell line (H520) showed no statistically significant difference, despite a relative decline in proliferation of about 10%. These results evidence an oncogenic role of *SLC22A18* and *SLC22A18AS* overexpression caused by epigenetic imbalances that promote tumor cell proliferation.

### 2.7. Reactome Pathway Analysis for the Imprinted SLC22A18 and SLC22A18AS Genes

**Figure 8:**
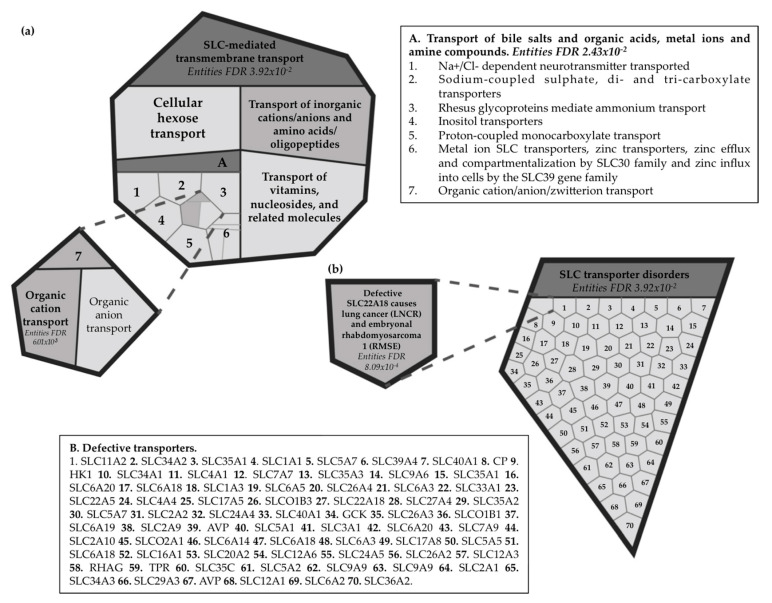
Representation of the enhanced reactome of the *SLC22A18* gene. (**a**) Solute carrier (SLC)-mediated transmembrane transport. (**b**) SLC transporter disorder. SLC: solute-carrier superfamily.

A bioinformatic approach was used to visualize the possible effects of the roles of *SLC22A18* and *SLC22A18AS* in the NSCLC context. *SLC22A18* plays a vital role as a membrane transporter. Under physiological conditions, SLC22A18, an SLC-mediated transmembrane transporter (false discovery rate, FDR = 3.92 × 10^−2^), has been specifically reported to be an organic cationic transporter (FDR = 6.01 × 10^−3^) based on a proton efflux (H^+^) antiport process (Figure 8a). On the other hand, defective SLC22A18 has been described in lung cancer (LNCR) and other types of tumors, such as embryonal rhabdomyosarcoma 1 (RMSE1) (FDR = 8.09 × 10^−4^) (Figure 8b). However, although two biological pathways have been identified to date for SLC22A18, none have been described for its antisense gene.

## 3. Discussion

In this study, we analyzed the methylation and expression status of the imprinted *SLC22A18* and *SLC22A18AS* genes in the context of NSCLC. We have shown here that both genes were in a hypomethylated status in NSCLC, which is a characteristic feature of the main histological subtypes, such as adenocarcinoma and SCC. *SLC22A18* and *SLC22A18AS* promoter DNA hypomethylation contributed to their overexpression. In addition, we have also provided evidence that the determination of *SLC22A18* and *SLC22A18AS* expression levels suggested a prognostic role for the time to first progression of the disease and overall survival of patients. 

The epigenetic instability of imprinted genes is receiving more attention in the cancer research community [19]. These genes are particularly vulnerable to having one specifically silenced parental allele and are frequently associated with both oncogenes and tumor suppressors [20]. Accordingly, a loss of genomic imprinting has been reported in a wide range of tumors, such as Wilms’ tumors, lung carcinomas, neuroblastomas, acute myeloblastic leukemias, rhabdomyosarcomas, and sporadic osteosarcomas [21]. In fact, many imprinted genes tend to be organized in large clusters (e.g., IGF2-H19, DLK1-DIO3 or C19MC), suggesting the potential involvement of higher order regulatory elements for these regions [14,22,23,24,25,26,27]. A better-characterized imprinted cluster in cancer is the chromosome 11p15.5 region. Specifically, in this region, *IGF2* overexpression was found to be caused by hypomethylation, which acts as a risk biomarker for colorectal carcinoma due to its implication in tumorigenesis promotion [28], as well as in different stages of progression and metastasis [29]. In lung cancer, loss of imprinting of the *IGF2* gene has also been reported as a growth-promoting alteration in lung adenocarcinoma [30]. However, there are other imprinted genes included in this same chromosomal region, 11p15.5, whose functions are unknown in cancer. Hence, we investigated the methylation pattern of two genes located in this imprinted region, *SLC22A18* and *SLC22A18AS*, to determine their possible effects in NSCLC patients.

*SLC22A18* and *SLC22A18AS* are examples of a sense/antisense imprinted gene pair; they are preferentially expressed based on the maternal allele and located in the imprinted region 11p15.5 together with *IGF2/IGF2AS* and *KCNQ1/KCNQ1OT1* [15,16]. To date, the *SLC22A18* methylation status is controversial because it depends on the type of sample analyzed. For example, Chu et al. showed high *SLC22A18* promoter methylation in glioma U251 cells. In addition, they found that aberrant promoter methylation contributed to low *SLC22A18* expression in glioma patients. Furthermore, the re-establishment of *SLC22A18* methylation status and the recovery of its expression levels in vitro inhibited cell proliferation by increasing the apoptosis rate and blocking cell growth and adhesion, while in vivo assays showed decreased tumor growth [31,32]. However, high *SLC22A18* expression levels have been reported in epithelial ovarian cancer [33]. Similar results were also obtained in pancreatic cancer, where there were significantly higher transcript levels of *SLC22A18* in tumors compared with those in nonneoplastic pancreatic tissue [34]. In this study, we report that the imprinted *SLC22A18* and *SLC22A18AS* genes were hypomethylated in NSCLC patients, regardless of the lung adenocarcinoma or SCC subtype. Moreover, we found an inverse association between the methylation status and the expression levels of *SLC22A18*. These results have also been corroborated in vitro, in which a DNA methylating agent (ademetionine) changed their pattern of expression in a panel of NSCLC cell lines. Therefore, DNA promoter methylation is a key mechanism for regulating the transcription of these genes. Distinct histological and molecular characteristics of both histological subtypes may be responsible for the observed changes at the level of gene promoter methylation of *SLC22A18* and *SLC22A18AS*, and consequently, their relationship with gene expression. With respect to cellular pathology, SCC normally arises in a main or lobar bronchus and lung adenocarcinoma is usually found in more peripheral parts of the lung. Some of these differences are due to smoking frequency and depth of inhalation [35]. Therefore, the impact of exposure to tobacco smoke on DNA methylation levels could make the difference between adenocarcinoma and SCC. Another factor that could be key is the differences in metabolism in both histological subtypes. It is well known how the energy-producing metabolic pathways are altered in tumor cells [36]. Even such differences can be observed according to tumor subtypes. For instance, Meijer et al. reported that the expression of two transporters, such as GLUT1 and MCT4, differs between adenocarcinoma and squamous cell carcinoma, and pointed to the hypoxic pattern as being responsible [37]. Therefore, the metabolomic features of each tumor subtype could be influenced by the proton antiporter activity of SLC22A18. On the other hand, little is known about *SLC22A18AS*, apart from the characterization done by Bajaj et al., who reported *SLC22A18AS* expression as DNA methylation-dependent but described no cellular function [15]. We also observed overexpression of *SLC22A18AS* in NSCLC mediated by promoter demethylation. In addition, expression of both sense/antisense-imprinted genes was positively correlated. In this particular direction, a functional Sp1 transcription factor has been reported as an activator of the *SLC22A18* and *SLC22A18AS* promoters [15,38]; this may support the notion that such a shared transcriptional regulatory mechanism may be potentially responsible for the positive correlation we observed between the two genes in our study.

Nevertheless, we did not find significant differences in the methylation levels of the *SLC22A18* and *SLC22A18AS* genes in the late and early stages. These results differ from those found by Lei et al., who reported that *SLC22A18* overexpression was markedly higher in NSCLC patients at a later TNM stage [39]. In addition, it has been proposed that miR-137 inhibits NSCLC aggressive progression through the regulation of the *SLC22A18* gene [40]. In our study, these differences were probably not observed because most of included patients were at the earliest stages of the disease. It would be necessary to carry out a new assay with a larger sample size and in which the different stages were better represented to evaluate the changes in the methylation status of the *SLC22A18* and *SLC22A18AS* genes and consequently their expression levels throughout disease progression. On the other hand, our results demonstrate that overexpression of the *SLC22A18* and *SLC22A18AS* genes was significantly associated with worsening progression for lung adenocarcinoma and SCC patients. Specifically, we found that high expression levels of both genes were associated with increased disease progression. This significant trend continued to be evident for the overall survival of patients with lung adenocarcinoma with higher *SLC22A18AS* expression levels. Therefore, there is high congruence between clinical TNM stage and *SLC22A18* expression [39,40]. It is clear that an advanced clinical stage is associated with worse prognoses for patients. Consistent with our research, the overexpression of *SLC22A18* was also correlated with a worse prognosis for people with ovarian and pancreatic tumors [33,34]. However, a finding opposite to our results on *SLC22A18* was reported for breast and colorectal tumors, where a lower expression for *SLC22A18* was correlated with a worse prognosis for these patients [41,42,43]. All these findings suggest that a possible role for *SLC22A18* as an oncogene or tumor suppressor gene varies considerably, depending on the type of tumor. *SLC22A18AS* seems to present the same effect as *SLC22A18* in NSCLC. However, no study has previously been carried out to analyze the role of this gene in this disease. Here, we provided evidence that one of the possible mechanisms that may explain the association of *SLC22A18* and *SLC22A18AS* expression with survival in lung cancer is through the promotion of cell proliferation. 

To explore the potential biological function of the *SLC22A18* and *SLC22A18AS* genes, we then visualized the results using a bioinformatics approach, showing that SLC22A18 plays a vital role as a SLC-mediated membrane transporter. Specifically, SLC22A18 acts as a transporter of organic cations based on proton antiport efflux. Thus, aberrations in its expression can alter cellular metabolism, growth and the response to drugs [44]. Defective SLC22A18 may be involved in different diseases, such as lung cancer. Recently, Ito et al. reported that *SLC22A18*-knockdown HepG2 cells had a decreased expression of IGFBP-1, which decreased cellular growth but increased invasiveness [45]. Loss of *SLC22A18* DNA methylation has been reported as a tumor suppressor in hepatocellular carcinoma [46]. However, more studies are needed to determine the functional mechanism of this gene in those tumors, where it acts as an oncogene, as our lung cancer results suggest. On the other hand, based on in vitro assays, *SLC22A18* has been suggested to be involved in an underlying mechanism of chemotherapy resistance [47]. It would be interesting to analyze *SLC22A18* expression in NSCLC patients based on the treatment that they received and/or on the development of a chemoresistance phenotype of the tumor. The same inquiry would apply for the *SLC22A18AS* gene, in which further studies are needed to unravel its role in lung tumorigenesis and the prognosis of the disease. Although a little headway has been recently made, Bajkowska et al. attributed to SLC22A18AS a key role in the epithelial–mesenchymal transition through the NPTNβ pathway in lung cancer cells, with higher cellular motility and invasion but not growth [48].

## 4. Materials and Methods

### 4.1. Patient and Sample Selection

This study was performed with a total of 126 subjects from Virgen del Rocio University Hospital (Seville, Spain). The subjects were separated into two independent cohorts. DNA methylation levels of the SLC22A18 and SLC22A18AS genes were evaluated in the first cohort (*N* = 70). Lung tissue specimens were collected from 47 patients with early-stage NSCLC and 23 without lung cancer who had undergone surgical procedures. These sets of samples constituted the study and control groups. SLC22A18 and SLC22A18AS expression changes were analyzed in a second cohort. This cohort comprised 56 NSCLC patients who had also received surgical treatment. From the ex vivo lung resection in both cohorts, tumor and nontumor paired tissues were acquired and frozen at −80 °C until further use. Prospective histologic evaluation of lung tissue samples showed that some patients had more advanced tumors (stage IIIB and IV). All participants were informed about the study, and their signed consent was obtained prior to their participation. Previously, the Ethical Committee of the Virgen del Rocio University Hospital approved the study protocol and the use of human samples (01690-N-17). The clinical description of both cohorts is summarized in Table 1.

### 4.2. Cell Culture and Treatments

The NSCLC cell lines used in this study were A549, H1781, H2009, H2228, H358, H520, H226, and Calu-1, which were obtained from ATCC. H3122 and H3255 were kindly provided by Dr. Ferrer and Dr. Ramirez, respectively. All cell lines were cultured in RPMI-1640 medium supplemented with 10% FBS as well as antibiotics and antimycotic agents to prevent contaminations. Cell lines were expanded and stored in liquid nitrogen. They were grown in monolayers, maintained at 37 °C in a humidified atmosphere with 5% CO_2_, and regularly tested for mycoplasma. 

Ademetionine (#S5109) was purchased from Selleckchem (Houston, TX, USA) and prepared on DMSO according to manufacturer’s procedure. Then, 3 × 10^5^ cells were seeded in 6-well plates and treated with 10% FBS fresh medium containing ademetionine 200 μM or DMSO as control. After 24 h, cells were collected, ready for their subsequent DNA and RNA extraction.

### 4.3. SiRNA Transfections

Cells were seed in suitable confluence in 96-well and 6-well plates for siRNA transfections. Cells were transfected with 10 nM of a siRNA to target SLC22A18 (#225917652; IDT, Coralville, IA, USA) and 20 nM of a siRNA to target SLC22A18AS (#s9907; Life Technologies, Carlsbad, CA, USA) using Lipofectamine RNAiMax reagent (#13778500; Thermo Fisher, Waltham, MA, USA) according to the manufacturer’s protocol. After 72 h of incubation, the cells were processed for further analysis. Silencer^TM^ Select Negative Control No. 1 siRNA (#4390843, Thermo Fisher) was used as control.

### 4.4. Cell Proliferation Assay 

Cell proliferation was assessed by Cell Counting Kit-8 (Dojindo Chemicals, Kumamoto, Japan) assay according to the manufacturer’s instructions. The cell proliferation assay was performed 72 h after siRNA transfection. Briefly, 10 μL of Cell Counting Kit-8 solution was added to each well, and incubated at 37 °C in a humidified atmosphere with 5% CO_2_ for 1 h. Absorbance at 450 nM was measured with an iMark^TM^ microplate reader (BioRad, Berkeley, CA, USA). Each assay was performed twice at least in duplicate. The percentage of cell number is shown relative to the Silencer^TM^ Select Negative Control.

### 4.5. Nucleic Acid Isolation

Nucleic acid was isolated from pulverized lung tissue using liquid nitrogen and cell line pellets. Genomic DNA was isolated by a QIAamp DNA mini kit (QIAGEN, Valencia, CA, USA) and fluorometrically quantified by a QuantiFluor dsDNA system (Promega, Madison, WI, USA) following the manufacturers’ protocols, and by NanoDrop 3000 (Thermo Fisher). Total RNA isolation was performed using the mirVana^TM^ miRNA isolation kit (INVITROGEN, Carlsbad, CA, USA) according to the manufacturer’s protocol. The isolated RNA was quantified with a NanoDrop 2000 (Thermo Fisher). The DNA and RNA samples were frozen at −20 and −80 °C, respectively, until further application.

### 4.6. Bisulfite Transformation

Briefly, 500 ng of genomic DNA was treated with sodium bisulfite following the EZ DNA MethylationTM protocol (EZ DNA, Zymo Research, Irvine, CA, USA). Transformed DNA was cleaned with a ZR-96 DNA Cleanup KitTM (EZ DNA, Zymo Research).

### 4.7. DNA Methylation Pattern of the SLC22A18 and SLC22A18AS Genes

The methylation profiles of the *SLC22A18* and *SLC22A18AS* genes were analyzed using Illumina Infinium Human Methylation 450 BeadChip (Illumina Inc. San Diego, CA, USA) as previously described [14]. Once DNA was transformed, it was prepared for the usual amplification, hybridization and imaging steps of the Illumina method. The derived intensity files were analyzed with Illumina GenomeStudio software. β-Scores were obtained from the fraction of total signal emitted by the methylation-specific probe or color channel.

### 4.8. Expression Levels of the SLC22A18 and SLC22A18AS Genes

For the expression analysis, 500 ng of total RNA was converted into cDNA using a High Capacity cDNA Reverse Transcription kit (Thermo Fisher). The reverse transcriptase reaction was performed through sequential incubations, according to the kit’s protocol. Then, 40 ng of cDNA was used for the expression analysis of the *SLC22A18* (probe Hs00945415_mL) and *SLC22A18AS* (probe Hs00757934_mL) genes by qPCR following the TaqMan Gene Expression Assay protocol (Thermo Fisher). The reaction was performed in a 7900 HT Fast Real-Time PCR system (Applied Biosystems, Foster City, CA, USA). The PCR mixture was incubated at 95 °C for 10 min followed by 40 cycles of 95 °C for 15 s and 60 °C for 60 s. All experiments were performed in triplicate.

The relative quantification of the *SLC22A18* and *SLC22A18AS* genes was performed using the 2^-∆C^_t_ method [49] to analyze the changes in the expression of the genes. For both genes of interest in each sample, ∆C_t_ was defined as the difference between the C_t_ (threshold cycle, the PCR cycle in which the fluorescence is higher than a threshold level) for the genes of interest versus the C_t_ for a reference gene (*β2-Microglobulin*, probe Hs99999907_mL). The C_t_ values were calculated with SDS software v2.4.1 (Applied Biosystems) using the automatic baseline setting and a threshold of 0.2.

### 4.9. Validation of the Expression Analysis for SLC22A18 and SLC22A18AS in the NSCLC Patients Using Public Databases

To confirm the robustness of the analysis framework, we explored the expression levels of *SLC22A18* and *SLC22A18AS* genes in NSCLC patients as reported in public databases, such as the Cancer Biomedical Informatics Grid (caBIG) [50], the Gene Expression Omnibus (GEO) [51], and The Cancer Genome Atlas (TCGA) (https://www.cancer.gov/tcga). Conventional searches using the keywords “lung”, “cancer”, “NSCLC”, and “survival” were used. The manual curation of the data was performed according to the microarray platform used (Affymetrix HG-U133A and HG-U133Plus 2.0). Finally, the expression levels of *SLC22A18* (probeset 204981_at) and *SLC22A18AS* (probeset 206097_at) were obtained from the following datasets: GSE3141, GSE8894, GSE14814, GSE19188, GSE29013, GSE31210 GSE37745 and GSE68465. The use of identical probe sets allowed us to measure both genes with similar accuracy within the same scale and dynamic range.

### 4.10. Data Analysis

The methylome data were analyzed by the RnBeads R package [51]. After performing quality control, the medium intensity of the probes was normalized with the SWAN method [52] and transformed to β values. The limma method was used to test the differential methylation [53]. The *p*-values were adjusted using the Benjamini–Hochberg method to ensure that the false discovery rate (FDR) was lower than 0.05. The CpG content and the methylation levels of both genes were visualized using the WashU Epigenome Browser v50.4.0 [54].

The expression data of the *SLC22A18* and *SLC22A18AS* genes were compared using the Mann–Whitney U test. Box-plot diagrams and heat maps were generated to visualize the expression changes between nontumor and tumor lung tissues or among histological subtypes. The Spearman’s correlation of both genes was found. All statistical analyses were performed with the SPSS statistical package (v23, IBM) and the *GraphPad QuickCalcs* (http://www.graphpad.com/quickcalcs/) (accessed June 2020). For the prognosis analyses, the Kaplan–Meier survival plots at 5-year to the time of first progression of the disease and the overall survival time were obtained using the Kaplan–Meier plotter website, where the unprocessed. CEL files of the caBIG, GEO and TCGA repositories were normalized by MAS5 in the R environment. Ten datasets are included in the Kaplan-Meier plotter website, namely GSE4573, GSE14814, GSE8894, GSE19188, GSE3141, GSE31210, caArray, TCGA, GSE29013, and GSE37745 [18]. The best performing threshold from lower and upper quartiles computed was used as cut-off for the definition of high and low expression of the *SLC22A18* and *SLC22A18AS* genes. First progression (FP) was defined as the time elapsed from the date of initiation of first-line treatment to the date of the first clinical evidence of disease progression. Overall survival (OS) was defined from the diagnosis to the date of death. P-values lower than 0.05 were considered significant. To explore biological pathways associated with *SLC22A18* expression, the reactome pathway database was consulted [55].

## 5. Conclusions

In this work, we confirmed that the imprinted *SLC22A18* and *SLC22A18AS* genes are overexpressed with a hypomethylated pattern of their promoter regions in NSCLC patients. These results reveal novel diagnostic CpG-based biomarkers for this disease. In addition, we have also shown that *SLC22A18* and *SLC22A18AS* expression levels are associated with clinical outcome; i.e., the overexpression of these genes was significantly correlated with worsening the progression of lung adenocarcinoma and SCC in patients. In addition, the expression of *SLC22A18AS* significantly predicted poor overall survival for patients with lung adenocarcinoma. Therefore, both genes also played a key role in the course of the disease, supporting their classification as oncogenes in NSCLC. On the other hand, *SLC22A18* is also involved in the cellular metabolism, growth and response to drugs of some tumors [44]. In the case of *SLC22A18AS*, no known biological function has been found [15]. Based on these results, further studies are needed to analyze the functions of both genes in lung cancers and to study their use as specific therapeutic targets.

## Figures and Tables

**Table 1:** Characteristics of the analyzed cohorts.

Characteristics	First cohort (*N* = 70)		Second cohort (*N* = 56)
	Study group (*N* = 47)	Control group (*N* = 23)	
**Age (years)**	67 (60–73)	35 (21–62)	69 (63–75]
**Gender**			
Male	76.6 (36)	87.0 (20)	82.1 (46)
Female	23.4 (11)	13.0 (3)	17.9 (10)
**Smoking status**			
Smokers	85.1 (40)	52.2 (12)	94.6 (53)
Nonsmokers	14.9 (7)	47.8 (11)	5.4 (3)
**Histology**			
Lung adenocarcinoma	57.4 (27)	-	50.0 (28)
Squamous cell lung carcinoma	42.6 (20)	-	50.0 (28)
**Staging**			
I	40.5 (19)	-	50.0 (28)
II	38.3 (18)	-	21.4 (12)
III–IV	21.2 (10)	-	17.9 (10)
**Subjects with COPD**	42.6 (20)	17.4 (4)	53.6 (30)

Continuous variables are expressed as the median (interquartile range (IQR)), and categorical variables are expressed as the percentage of cases (number of cases). In both cohorts, a patient who met at least one of the following conditions was considered to be a smoker: a regular smoker or an ex-smoker who smoked for more than 15 years or for less than 15 years but with a package/year ratio higher than 20.

## Supplementary Materials

The following are available online at https://www.mdpi.com/2072-6694/12/8/2075/s1, Figure S1: Methylation status of CpG islands (log2 ratio) in the promoter regions of the *SLC22A18* (a) and *SLC22A18AS* (b) genes in NSCLC patients; Figure S2: Heat map comparison of the differential expression of *SLC22A18* and *SLC22A18AS* according to 8 public lung cancer datasets (GSE3141, GSE8894, GSE14814, GSE19188, GSE29013, GSE31210 GSE37745 and GSE68465).

## Abbreviations

NSCLC	Non-small cell lung cancer	SCLC	Small cell lung cancer
SCC	Squamous cell carcinoma	ADC	Adenocarcinoma
SLC22A18	Solute-carrier family 22 member 18	TCGA	The Cancer Genome Atlas
SLC22A18AS	Solute-carrier family 22 member 18 antisense	HR	Hazard ratio
CI	Confidence interval	FDR	False discovery rate
NT	Nontumoral lung tissue	DMSO	Dimethyl Sulfoxide
